# Targeting Inhibin Enhances Wagyu Oocyte Competence and Embryo Quality: A Comparative Study of In Vivo Immunization and In Vitro Antibody Supplementation

**DOI:** 10.3390/antiox15040414

**Published:** 2026-03-26

**Authors:** Jingyu Ren, Fuhan Liu, Gang Liu, Biao Wang, Jie Zhu, Yongbin Liu, Yanfeng Dai

**Affiliations:** 1 Key Laboratory of Herbivorous Livestock Reproductive Regulation, National Sheep Genetic Evaluation Center, Inner Mongolia University, Hohhot 010030, China; 31608050@mail.imu.edu.cn (J.R.);; 2 Beijing Jingwa Agricultural Science & Technology Innovation Center, Beijing 101205, China; 3 Clinical Medicine Research Center, Affiliated Hospital of Inner Mongolia Medical University, 1 Tongdao North Street, Hohhot 010050, China; 4 Department of Animal Genetics, Breeding, and Reproduction, College of Animal Science, Inner Mongolia Agricultural University, Hohhot 010070, China; 5 Animal Husbandry Institute, Inner Mongolia Academy of Agricultural & Animal Husbandry Sciences, No. 22 Zhaowuda Road, Hohhot 010031, China

**Keywords:** Wagyu cattle, inhibin, OPU-IVP, oxidative stress, mitochondria, oocyte competence

## Abstract

High-efficiency Ovum Pick-Up (OPU) and in vitro embryo production (IVP) are critical for the genetic improvement of high-value Wagyu cattle. However, oxidative stress and mitochondrial dysfunction during oocyte maturation remain major bottlenecks limiting blastocyst yield. This study investigated the role of inhibin in Wagyu oocyte competence through two independent proof-of-concept approaches. In the in vivo active immunization model, thirty Wagyu donors were immunized with a recombinant inhibin protein (INHA group), resulting in a significant increase in the number of recovered cumulus–oocyte complexes (COCs) (461 vs. 279, *p* < 0.05) and the proportion of high-quality oocytes compared to controls. Oocytes from the INHA group exhibited improved cytoplasmic maturation and mitochondrial function, characterized by higher membrane potential (ΔΨm, JC-1 ratio: 1.55 ± 0.06 vs. 0.83 ± 0.08, *p* < 0.05), elevated ATP content (2.35 ± 0.07 vs. 1.63 ± 0.03 pmol/oocyte, *p* < 0.05), and increased NADPH levels. Furthermore, the INHA group showed significantly reduced reactive oxygen species (ROS) accumulation and an increased GSH/GSSG ratio (8.48 ± 0.18 vs. 6.25 ± 0.09, *p* < 0.05), indicating restored redox homeostasis. Independently, in the in vitro anti-inhibin antibody (AIA) supplementation model, AIA supplementation during oocyte maturation significantly improved the nuclear maturation rate (92.96% ± 1.04%), blastocyst formation rate (56.63% ± 2.36%), and total cell number compared to controls (*p* < 0.05). Notably, AIA-derived blastocysts achieved a significantly higher pregnancy rate (78.65% ± 1.57%) following transfer. Collectively, these findings demonstrate that targeting inhibin mitigates oxidative injury and stabilizes mitochondrial bioenergetics, providing two distinct, physiology-based strategies for optimizing Wagyu oocyte yield and embryo production.

## 1. Introduction

The global demand for Japanese Black (Wagyu) beef, renowned for its intense marbling and unique organoleptic properties, has surged in recent years. To meet this demand and accelerate the dissemination of elite genetics, OPU and IVP have replaced traditional superovulation as the gold standard in commercial breeding programs [1,2]. OPU allows for the retrieval of oocytes from donors regardless of their reproductive cycle stage, theoretically maximizing the reproductive potential of high-genetic-merit cows. However, the widespread application of this technology is currently hampered by a critical bottleneck: the heterogeneity of the retrieved oocyte population. To obtain a cohort of antral follicles with similar intrinsic quality, a number of strategies, such as the mechanical ablation of all antral follicles followed by hormonal stimulation, have been widely adopted. However, standard OPU often indiscriminately harvests COCs from a cohort largely composed of subordinate or static follicles [3]. These oocytes often exhibit “competence failure,” characterized by incomplete cytoplasmic maturation, impaired metabolic activity, and poor resilience to in vitro handling, resulting in blastocyst rates that rarely exceed 30–40% [4,5].

The transition from the physiological ovarian environment to in vitro culture conditions imposes substantial stress on the oocyte. Among the various stressors, oxidative stress—defined as an imbalance between the production of reactive oxygen species (ROS) and the capacity of intrinsic antioxidant defense systems—is widely recognized as the primary mediator of developmental arrest [6,7]. Excessive ROS accumulation triggers a cascade of deleterious events, including lipid peroxidation of cellular membranes, DNA strand breaks, and enzyme inactivation [8].

More critically, ROS targets the mitochondria, the “powerhouse” of the oocyte. Functional mitochondria are essential for generating adenosine triphosphate (ATP) via oxidative phosphorylation to fuel meiotic spindle assembly and early cleavage [9]. Oxidative injury leads to the opening of mitochondrial permeability transition pores, depolarization of the mitochondrial membrane potential (ΔΨm), and subsequent depletion of ATP stores [10]. Previous studies have demonstrated that bolstering the endogenous antioxidant capacity—such as through targeted metabolic supplementation—can effectively preserve mitochondrial integrity and rescue oocyte developmental competence under stress conditions [11,12,13]. Therefore, identifying physiological regulators that can inherently bolster mitochondrial function is a priority for optimizing IVP systems.

In the quest to improve oocyte quality, inhibin, a dimeric glycoprotein of the transforming growth factor-β (TGF-β) superfamily, has emerged as a key target. Classically, inhibin is characterized as a potent endocrine suppressor that inhibits follicle-stimulating hormone (FSH) secretion from the pituitary gland via a negative feedback loop [14]. Active immunization against inhibin has been extensively utilized to neutralize this suppression, thereby elevating circulating FSH levels and promoting the recruitment of a larger cohort of follicles [15,16].

However, the role of inhibin extends beyond the pituitary–gonadal axis. Within the ovary, inhibin is primarily secreted by granulosa cells in growing follicles and acts as a crucial paracrine/autocrine regulator. It functions as a potent antagonist to Activins and Bone Morphogenetic Proteins (BMPs)—factors that are indispensable for granulosa cell proliferation, steroidogenesis, and oocyte maturation [14]. High intrafollicular inhibin concentrations, typical of subordinate or atretic follicles, effectively block Activin signaling by competing for the Type II receptor (ActRII) or recruiting the co-receptor betaglycan, thereby suppressing the SMAD2/3 signaling pathway [17].

Despite the well-established role of inhibin in follicular dynamics, its direct impact on the “intrinsic quality” of the oocyte—specifically its metabolic and oxidative status—remains poorly understood. Emerging evidence suggests a link between TGF-β superfamily signaling and cellular metabolism. For instance, Activin signaling has been shown to promote glucose uptake and mitochondrial biogenesis in various cell types [18]. Conversely, we hypothesized that the local accumulation of inhibin in the follicular fluid might exert a “metabolic brake” on the oocyte–cumulus complex, restricting mitochondrial activity and rendering the oocyte more susceptible to oxidative stress. If this hypothesis holds, neutralizing inhibin could not only increase follicle numbers but also “unleash” the metabolic potential of the oocytes.

Based on this rationale, we investigated the role of inhibin in Wagyu oocyte competence through two independent proof-of-concept experimental models. The first approach utilized in vivo active immunization of Wagyu donors to evaluate OPU recovery efficiency and COC morphological grades. This was followed by elucidating the initial cellular mechanisms of the retrieved oocytes, specifically by assessing cytoplasmic maturation, mitochondrial bioenergetics (distribution, ΔΨm, ATP content), and redox homeostasis (ROS, GSH/GSSG). The second approach utilized an independent in vitro maturation system supplemented with AIA using abattoir-derived ovaries. This in vitro step aimed to neutralize locally secreted inhibin in the culture microenvironment, serving as a mechanistic model to evaluate downstream developmental competence through blastocyst quality assessment, lineage specification, and a field embryo transfer trial under “super-physiologic” conditions that favor Activin-mediated metabolic support [19]. Our findings provide novel insights into the interplay between inhibin signaling and oocyte bioenergetics, offering two distinct, physiology-based strategies to overcome efficiency barriers in bovine embryo production.

## 2. Materials and Methods

### 2.1. Ethics Statement and Reagents

All in vivo experimental procedures involving live Wagyu cattle, including immunization, ovum pick-up (OPU), and embryo transfer, were formally reviewed and approved by the Experimental Animal Ethics Committee of Inner Mongolia University (Ethical Approval No. 2024/073). All in vitro embryo production experiments utilized abattoir-derived ovaries, which are by-products of the food industry, and thus did not require additional specific live-animal ethics approval. All procedures were conducted in strict accordance with relevant national and institutional guidelines for animal welfare. Unless otherwise stated, all chemicals and reagents were obtained from Sigma-Aldrich (St. Louis, MO, USA).

Part 1: In Vivo Immunization and Oocyte Quality Assessment.

### 2.2. Experimental Design 1: In Vivo Immunization Strategy

To evaluate the physiological effects of active immunization against inhibin on oocyte competence, sixty healthy, cycling Japanese Black (Wagyu) donors (aged 3–5 years) were randomly assigned to two groups: (1) Immunized Group (INH, n = 30): Donors were actively immunized with the recombinant inhibin protein to induce neutralizing antibodies. (2) Control Group (Control, n = 30): Donors received an equivalent volume of physiological saline (with adjuvant). The primary endpoint was the efficiency of follicular recruitment (OPU yield), and the secondary endpoints were the physiological status of retrieved oocytes.

To strictly avoid confounding factors, OPU-derived oocytes from these live donors were dedicated exclusively to terminal physiological and cellular assays and were not subjected to any downstream in vitro embryo culture.

### 2.3. Preparation of Inhibin Antigen and Immunization Protocol

The recombinant bovine inhibin α-subunit protein (residues 1–134) was expressed in Escherichia coli BL21 (DE3) utilizing the pET-28a vector system. The protein was purified via Ni-NTA affinity chromatography followed by strict endotoxin removal to ensure levels were <0.1 EU/mL [20,21]. For immunization, 1 mg of the purified protein was emulsified with Freund’s complete adjuvant (FCA; F5881, Sigma-Aldrich, St. Louis, MO, USA) for the primary injection and Freund’s incomplete adjuvant (FIA; F5506, Sigma-Aldrich, St. Louis, MO, USA) for the booster injections. Donors in the INHA group received the emulsified antigen strictly via intramuscular injection on Days 0, 21, and 42 to ensure consistency.

### 2.4. OPU and Oocyte Collection

OPU sessions were initiated one week after the second booster and performed weekly for three consecutive weeks. Donors were sedated, and follicular aspiration was performed using a portable B-mode ultrasound scanner (Model XYZ123, GE Healthcare, Chicago, IL, USA) equipped with a 5.0–7.5 MHz transvaginal convex probe and a 17-gauge needle guided by a vacuum pump (flow rate: 15 mL/min; negative pressure: 80–100 mmHg). Follicular fluid was collected into 50-mL conical tubes containing phosphate-buffered saline (PBS; 14190250, Thermo Fisher, Beijing, China) supplemented with 10 IU/mL heparin and 1% fetal bovine serum (FBS; 30044333, Thermo Fisher, Shanghai, China). Retrieved cumulus–oocyte complexes (COCs) were washed and graded. Only Grade A and B COCs (compact cumulus layers and uniform cytoplasm) were used for subsequent assays.

### 2.5. Assessment of Oocyte Cytoplasmic Maturation (Cortical Granules)

To evaluate cytoplasmic maturation, cortical granule (CG) distribution was assessed using fluorescein isothiocyanate-labeled Lens culinaris agglutinin (LCA-FITC; L9137, Sigma-Aldrich, St. Louis, MO, USA). Denuded oocytes were fixed in 4% paraformaldehyde (P1110, Solarbio, Beijing, China) for 30 min and permeabilized with 0.5% Triton X-100 (T8200, Solarbio, Beijing, China) for 15 min. After blocking with 1% BSA (A3803, Sigma-Aldrich, St. Louis, MO, USA) for 1 h, oocytes were incubated with 100 μg/mL LCA-FITC for 30 min at 38.5 °C in the dark. Nuclei were counterstained with Hoechst 33342 (C1022, Beyotime, Shanghai, China).

Samples were mounted and observed under a confocal laser scanning microscope (A1R, Nikon, Tokyo, Japan). All fluorescence images were acquired under strictly identical confocal microscope settings. Image acquisition was deliberately performed within the linear dynamic range to avoid any signal saturation. All subsequent quantitative analyses of fluorescence intensity were conducted exclusively on the raw, non-adjusted images to ensure accuracy.

A dedicated subset of at least 30 oocytes per group in total (distributed across three independent biological replicates) was randomly allocated for these assays. To strictly avoid pseudoreplication, statistical analyses were performed exclusively on the replicate-level means (n = 3).

### 2.6. Evaluation of Mitochondrial Distribution and Membrane Potential

Mitochondrial function was evaluated using specific fluorescent probes.

Mitochondrial Distribution: Freshly retrieved oocytes were incubated with 200 nM MitoTracker Red CMXRos (C1049, Beyotime, Shanghai, China) in M199 medium for 30 min at 38.5 °C in the dark. Mitochondrial Membrane Potential (ΔΨm): Oocytes were incubated with 2 μM JC-1 (C2006, Beyotime, Shanghai, China) for 30 min at 38.5 °C. The ratio of red (J-aggregates, high potential) to green (monomers, low potential) fluorescence intensity was calculated to quantify ΔΨm.

For all relevant staining (MitoTracker and JC-1), fluorescence intensity was quantified across the entire ooplasm (deliberately excluding the zona pellucida and background) using ImageJ software (version 1.54, NIH, Bethesda, MD, USA).

A dedicated subset of at least 30 oocytes per group in total (distributed across three independent biological replicates) was randomly allocated for these assays. To strictly avoid pseudoreplication, statistical analyses were performed exclusively on the replicate-level means (n = 3).

### 2.7. Quantification of Intracellular ROS and Metabolites

ROS Levels: Intracellular reactive oxygen species were detected using 2′,7′-dichlorodihydrofluorescein diacetate (DCFH-DA; S0033, Beyotime, Shanghai, China). Oocytes were incubated with 10 μM DCFH-DA in PBS for 20 min at 38.5 °C. Green fluorescence was captured immediately by confocal microscopy.

Metabolite Assays: Intracellular contents of ATP, NADPH, and Glutathione (GSH/GSSG) were measured using commercial assay kits (S0026 for ATP, S0179 for NADPH, S0053 for GSH/GSSG, Beyotime, Shanghai, China) according to the manufacturer’s instructions. Bioluminescence or absorbance was measured using a microplate reader (Model SpectraMax i3x, Molecular Devices, San Jose, CA, USA).

A dedicated subset of at least 30 oocytes per group in total (distributed across three independent biological replicates) was randomly allocated for these assays. To strictly avoid pseudoreplication, statistical analyses were performed exclusively on the replicate-level means (n = 3).

Part 2: In vitro Modulation and Embryo Production

### 2.8. Experimental Design 2: In Vitro Modulation System

To investigate whether neutralizing inhibin in vitro mimics the beneficial effects of immunization, a separate experiment was conducted using COCs derived from Slaughterhouse ovaries. COCs were randomly allocated to four treatment groups during IVM: (1) Control Group: Cultured in standard IVM medium. (2) AIA Group: Supplemented with Anti-Inhibin Antibody (ab171107, Abcam, Cambridge, UK; 1 μg/mL). A higher initial number of COCs was specifically allocated to this experimental group to ensure robust statistical power for the extensive downstream destructive assays. (3) AIA + INHA Group: Supplemented with AIA and Recombinant Inhibin A protein (ab85308, Abcam, Cambridge, UK) to verify antibody specificity. (4) IgG Group: Supplemented with non-specific IgG isotype (ab37415, Abcam, Cambridge, UK; 1 μg/mL).

### 2.9. In Vitro Maturation (IVM)

COCs were washed and cultured in maturation medium consisting of TCM-199 (11150067, Thermo Fisher, Waltham, MA, USA) supplemented with 10% FBS (30044333, Thermo Fisher, Waltham, MA, USA; the exact same batch was strictly used for all in vitro experiments to ensure identical baseline conditions), 1 μg/mL estradiol (E8875, Sigma-Aldrich, St. Louis, MO, USA), 0.5 μg/mL FSH (Sansheng, Ningbo, China), 10 ng/mL EGF (E9644, Sigma-Aldrich, St. Louis, MO, USA), and 0.1 mM cysteine (M9768, Sigma-Aldrich, St. Louis, MO, USA). The specific treatments (AIA, INHA, IgG) were added to the medium at the onset of culture. Maturation was performed in 4-well plates (Nunc, Roskilde, Denmark) at 38.5 °C under 5% CO_2_ in humidified air for 22–24 h.

### 2.10. In Vitro Fertilization (IVF) and Embryo Culture (IVC)

Frozen-thawed semen from a single fertile Wagyu bull was used. Sperm were selected using a Percoll gradient (P1644, Sigma-Aldrich, St. Louis, MO, USA) and adjusted to 1 × 10^6^ sperm/mL in IVF medium (Tyrode’s lactate solution supplemented with BSA, heparin, and hypotaurine). Matured oocytes were co-incubated with sperm for 6–8 h. Presumptive zygotes were denuded and cultured in Synthetic Oviductal Fluid (SOF) medium supplemented with 0.8% BSA (A3803, Sigma-Aldrich, St. Louis, MO, USA) under mineral oil at 38.5 °C in a low-oxygen atmosphere (5% O_2_, 5% CO_2_, 90% N_2_). Cleavage and blastocyst rates were recorded on Day 2 and Day 7, respectively.

### 2.11. Evaluation of Blastocyst Quality (TUNEL and Lineage)

Day 7 blastocysts were fixed in 4% paraformaldehyde and subjected to:

Apoptosis Assay: DNA fragmentation was detected using the In Situ Cell Death Detection Kit (TUNEL; C1091, Beyotime, Shanghai, China) following the manufacturer’s protocol [12].

Lineage Immunofluorescence: Blastocysts were incubated overnight at 4 °C with primary antibodies against SOX2 (1:500; ab137385, Abcam, Shanghai, China) and CDX2 (1:500; ab76541, Abcam, Shanghai, China). After washing, they were incubated with Alexa Fluor 555-conjugated (ab150074, Abcam, Shanghai, China) and Alexa Fluor 488-conjugated secondary antibodies for 1 h at room temperature.

Nuclei were counterstained with Hoechst 33342. Images were captured by confocal microscopy to analyze the Total Cell Number, Apoptotic Index, and ICM/TE ratio.

### 2.12. Embryo Vitrification and Transfer

Blastocysts from the four groups were vitrified using the Cryotop method (Kitazato, Tokyo, Japan). For the field trial, strict recipient controls were applied to minimize variability. Recipients were restricted to healthy, nulliparous heifers (14–16 months old) with synchronized estrus and a high-quality corpus luteum (>15 mm) on Day 7 of the estrous cycle. Qualified recipients were randomly allocated and strictly balanced across the three independent IVP replicates. All vitrified-warmed blastocysts were transferred non-surgically into the uterine horn ipsilateral to the corpus luteum by the same experienced technician at a single farm. Pregnancy was diagnosed by rectal palpation and ultrasonography 60 days post-transfer.

### 2.13. Statistical Analysis

All experiments were replicated at least three times. Data analysis was performed using SPSS 25.0 (IBM Corp., Armonk, NY, USA). Prior to parametric analysis, all datasets were confirmed to meet the assumptions of normality using the Shapiro–Wilk test and variance homogeneity using Levene’s test. To strictly avoid pseudoreplication, the experimental units were explicitly defined: for in vivo OPU and donor data, the independent donor cow (n = 30) was defined as the statistical unit; for all in vitro IVP parameters, blastocyst quality assessments (Total Cell Number, ICM/TE ratio, Apoptotic Index), and all oocyte-level cellular assays (including mitochondrial function, ROS, and metabolic measurements), statistical analysis was performed on replicate-level means, with the independent biological replicate defined as the experimental unit (n = 3). Differences between the two groups (in vivo study) were evaluated using the independent-samples *t*-test. Differences among multiple groups (in vitro study) were evaluated using one-way ANOVA followed by Tukey’s post hoc test. Differences in pregnancy rates (binary categorical outcomes) were analyzed using the Chi-square test. All data are expressed as mean ± SEM, and exact *p*-values are reported where applicable (*p* < 0.05 was considered statistically significant).

## 3. Results

### 3.1. Active Immunization Against Inhibin Increases Oocyte Recovery Efficiency

To evaluate the efficacy of the in vivo immunomodulation strategy on ovarian response, we monitored the oocyte recovery efficiency during OPU sessions. Following the immunization protocol, the INHA group exhibited a robust response in terms of oocyte yield. As shown in Table 1, the total number of COCs recovered per donor was significantly elevated in the INHA group compared to the Control group (461 vs. 279, *p* = 0.002). This substantial increase indicates that active immunization effectively enhances the yield of oocytes available for aspiration.

### 3.2. Improvement of Oocyte Morphological Quality and Grading

Beyond numerical increments, we further assessed whether the retrieved oocytes possessed superior morphological quality. COCs were classified into four grades (A–D) based on the compactness of cumulus layers and the homogeneity of the ooplasm. The INHA group yielded a significantly higher proportion of high-quality COCs compared to the Control group. Specifically, the percentage of Grade A COCs in the INHA group was 45.66 ± 1.15%, significantly higher than the 34.99 ± 1.25% observed in the Control (*p* = 0.003). Similarly, Grade B COCs were more frequent in the INHA group (33.89 ± 1.17% vs. 25.71 ± 1.62%, *p* = 0.015). Conversely, the proportions of low-quality Grade C (11.98 ± 1.23% vs. 20.26 ± 2.42%, *p* = 0.038) and D (8.47 ± 0.29% vs. 29.04 ± 0.48%, *p* ≤ 0.001) oocytes were significantly reduced in the INHA group, suggesting that the “inhibin-neutralized” follicular microenvironment supports healthier follicular development and reduces atresia (Table 1).

### 3.3. Enhancement of Oocyte Cytoplasmic Maturation (Cortical Granules)

**Figure 1:**
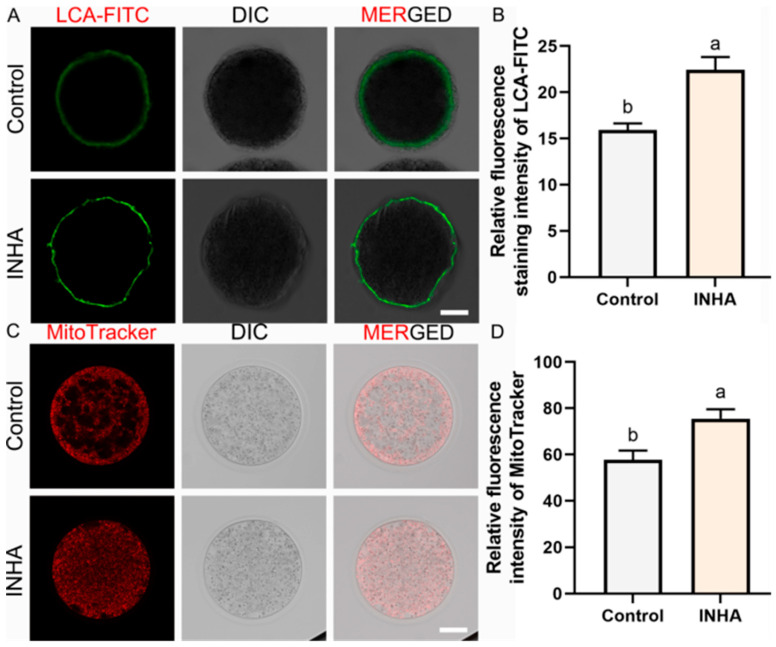
Active immunization against inhibin improves oocyte cytoplasmic maturation and mitochondrial distribution. (**A**). Representative images of cortical granules (CGs). LCA-FITC: Green fluorescence labeling, CGs. MERGE: Merged results of LCA-FITC fluorescence and DIC. Scale bar = 50 μm. (**B**). Quantitative analysis of CG fluorescence intensity. (**C**). Representative images of mitochondrial distribution. MitoTracker Red: Red fluorescence labeling, active mitochondria. MERGE: Merged results of MitoTracker fluorescence and DIC. Scale bar = 50 μm. (**D**). Quantification of mitochondrial fluorescence intensity. Note: Different lowercase letters (a, b) above the bars indicate significant differences between groups (*p* < 0.05). Data are presented as the mean ± SEM of three independent biological replicates. A total of at least 30 oocytes per group were assessed across the replicates, and the replicate-level means were used for statistical analysis.

Cytoplasmic maturation is a critical determinant of fertilization potential. We evaluated this by analyzing the distribution of cortical granules (CGs) using LCA-FITC staining. In oocytes derived from the INHA group, CGs displayed a continuous, uniform subcortical distribution (Figure 1A), which is a hallmark of fully mature ooplasm. In contrast, oocytes from the Control group frequently exhibited discontinuous, clustered, or inward-migrating CG patterns, indicative of incomplete maturation. Quantitative analysis of fluorescence intensity confirmed this observation, with the INHA group showing a significantly higher intensity (22.45 ± 0.80) compared to the Control group (15.92 ± 0.40, *p* = 0.002) (Figure 1B).

### 3.4. Optimization of Mitochondrial Distribution Patterns

Mitochondria organization is closely linked to oocyte competence. We utilized MitoTracker Red staining to visualize mitochondrial distribution. Oocytes from the INHA group exhibited a “healthy” distribution pattern, characterized by a fine, homogeneous reticular network throughout the cytoplasm (Figure 1C). This pattern facilitates efficient energy delivery to all cellular compartments. Conversely, oocytes from the Control group often displayed aggregated or peripherally localized mitochondria, a sign of organelle dysfunction or stress. The relative fluorescence intensity of mitochondria was significantly higher in the INHA group (75.49 ± 2.30) compared to the Control (57.67 ± 2.35, *p* = 0.006) (Figure 1D), suggesting an increase in mitochondrial mass or activity.

### 3.5. Restoration of Mitochondrial Membrane Potential

**Figure 2:**
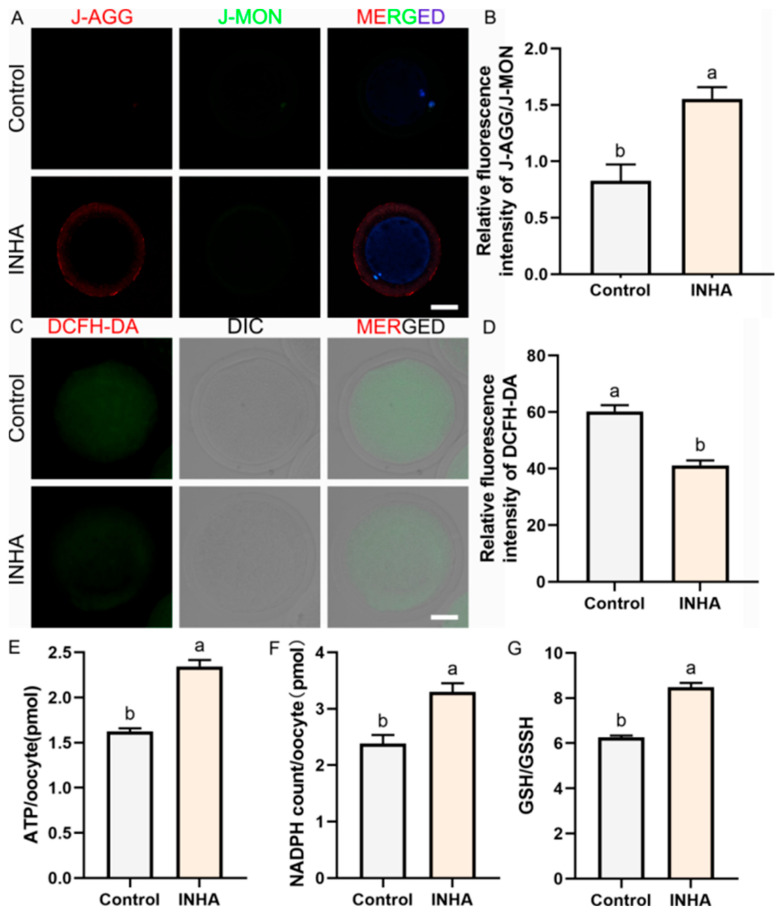
Enhancement of mitochondrial function and redox homeostasis in oocytes derived from immunized donors. (**A**). Assessment of mitochondrial membrane potential (ΔΨm) using JC-1 staining. JC-1: Red fluorescence indicates high potential (J-AGG), and green indicates low potential (J-MON). Scale bar = 50 μm. (**B**). Relative fluorescence ratio of J-AGG/J-MON (Mitochondrial membrane potential index). (**C**). Representative images of intracellular reactive oxygen species (ROS) levels. DCFH-DA: Green fluorescence indicating oxidative stress. MERGE: Merged results of DCFH-DA fluorescence and DIC. Scale bar = 50 μm. (**D**). Relative fluorescence intensity of ROS. (**E**). Intracellular ATP content analysis. (**F**). Intracellular NADPH level analysis. (**G**). The ratio of reduced glutathione to oxidized glutathione (GSH/GSSG). Note: Different lowercase letters (a, b) above the bars indicate significant differences between groups (*p* < 0.05). Data are presented as the mean ± SEM of three independent biological replicates. A total of at least 30 oocytes per group were assessed across the replicates, and the replicate-level means were used for statistical analysis.

To further investigate mitochondrial function, we measured the mitochondrial membrane potential (ΔΨm) using the JC-1 probe. High ΔΨm is essential for ATP production. As shown in Figure 2A, oocytes from the INHA group exhibited strong red fluorescence (J-aggregates), indicating high polarization. In contrast, Control oocytes showed predominantly green fluorescence (monomers), reflecting depolarized membranes. The Red/Green fluorescence ratio in the INHA group was 1.55 ± 0.06, which was nearly double that of the Control group (0.83 ± 0.08, *p* = 0.002) (Figure 2B). This result confirms that active immunization preserves mitochondrial bioenergetics in retrieved oocytes.

### 3.6. Alleviation of Intracellular Reactive Oxygen Species (ROS)

Oxidative stress is a major culprit in oocyte quality decline. We assessed intracellular ROS levels using DCFH-DA staining. Oocytes from the INHA group showed significantly reduced green fluorescence signals compared to the Control group (Figure 2C). Quantitative analysis revealed that the relative ROS levels in the INHA group (41.20 ± 1.00) were significantly lower than those in the Control group (60.25 ± 1.23, *p* = 0.0003) (Figure 2D). This data indicates that the in vivo neutralization of inhibin creates a low-oxidative-stress environment within the follicle, protecting the oocyte from oxidative damage.

### 3.7. Re-Establishment of Intracellular Metabolic and Redox Homeostasis

To understand the metabolic basis of the improved oocyte quality, we quantified key intracellular metabolites. Consistent with the restored mitochondrial function, ATP content was significantly elevated in the INHA group (2.35 ± 0.07 pmol/oocyte) compared to the Control (1.63 ± 0.03 pmol/oocyte, *p* = 0.001) (Figure 2E). Additionally, the INHA group exhibited higher levels of NADPH (3.30 ± 0.09 pmol/oocyte vs. 2.38 ± 0.09 pmol/oocyte, *p* = 0.002) (Figure 2F). Crucially, the ratio of reduced to oxidized glutathione (GSH/GSSG), a key indicator of antioxidant capacity, was significantly higher in the INHA group (8.48 ± 0.18) than in the Control (6.25 ± 0.09, *p* ≤ 0.001) (Figure 2G). These findings demonstrate a systemic improvement in cellular energy status and redox balance.

### 3.8. Anti-Inhibin Antibody Supplementation Enhances IVM and IVP Efficiency

To determine whether the benefits of inhibin neutralization could be replicated in vitro, we supplemented the IVM system with Anti-Inhibin Antibody (AIA). As presented in Table 2, the AIA group achieved the highest nuclear maturation rate (92.96 ± 1.04%), significantly surpassing the Control (83.72 ± 1.60%, *p* = 0.001), AIA + INHA (82.49 ± 1.61%, *p* ≤ 0.001), and IgG (82.76 ± 0.88%, *p* ≤ 0.001) groups.

This advantage persisted through subsequent development. The cleavage rate in the AIA group (86.32 ± 2.11%) was significantly higher than in the Control (75.16 ± 0.29%, *p* ≤ 0.001). Most importantly, the blastocyst formation rate in the AIA group reached 56.63 ± 2.36%, significantly outperforming the Control group (44.07 ± 0.85%, *p* ≤ 0.001). The specificity of the antibody was confirmed, as the addition of exogenous inhibin protein (AIA + INHA group) nullified these improvements.

### 3.9. Improvement of Blastocyst Quality, Lineage Allocation, and Pregnancy Outcomes

**Figure 3:**
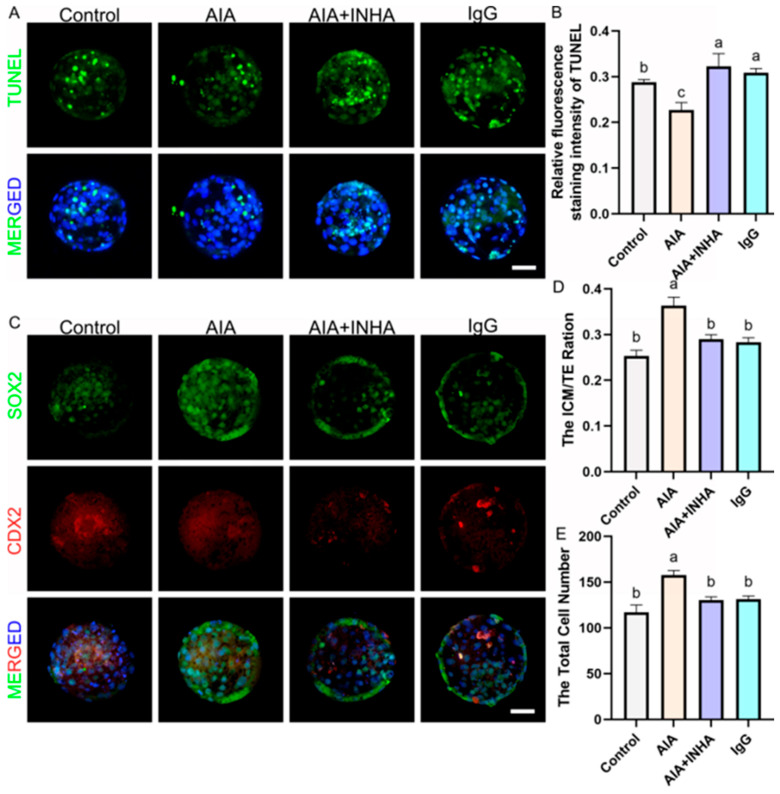
Anti-inhibin antibody supplementation enhances blastocyst quality and regulates lineage specification. (**A**). Representative images of apoptosis detected by TUNEL staining. TUNEL: Green fluorescence labeling of fragmented DNA, indicating apoptotic cells. MERGE: Merged results of TUNEL fluorescence and Hoechst 33342 staining (blue). Scale bar = 50 μm. (**B**). Relative fluorescence intensity of TUNEL staining (Apoptotic cell ratio). (**C**). Representative images of lineage specification. SOX2: Green fluorescence indicating the Inner Cell Mass (ICM). CDX2: Red fluorescence indicating the Trophectoderm (TE). MERGE: Merged results of SOX2/CDX2 fluorescence and Hoechst 33342 staining (blue, for all nuclei). Scale bar = 50 μm. (**D**,**E**). Quantitative analysis of blastocyst quality parameters (Total cell number, ICM cell number, and TE cell number). Note: Different lowercase letters in the bar charts indicate significant differences between groups (*p* < 0.05).

Finally, we evaluated the quality and viability of the resulting embryos. Blastocysts derived from the AIA group possessed significantly higher total cell numbers (151.4 ± 6.2) compared to the Control (117.6 ± 5.8, *p* ≤ 0.001). The Inner Cell Mass (ICM) to Trophectoderm (TE) ratio was also significantly improved in the AIA group (0.36 ± 0.02 vs. 0.28 ± 0.03, *p* ≤ 0.001), indicating enhanced developmental potential. Furthermore, the TUNEL assay revealed a significantly lower apoptotic index in the AIA group (3.8 ± 0.5%) compared to the Control (7.2 ± 0.8%, *p* = 0.0023) (Figure 3).

In the field trial, a total of 452 synchronized recipient heifers were utilized across the groups over three independent biological replicates. Specifically, across these three replicates, the total number of recipients allocated to the Control, AIA, AIA + INHA, and IgG groups was 80, 186, 93, and 93, respectively. While post-warming survival rates were similar across groups, the pregnancy rate following the single-embryo transfer of vitrified blastocysts was significantly higher in the AIA group (78.65% ± 1.57%) compared to the Control (67.26% ± 2.33%, *p* = 0.0024). While pregnancy is a binary and multifactorial outcome, and advanced mixed-effects modeling was not applied, these results provide robust descriptive associations supporting the enhanced post-thaw viability of AIA-derived embryos (Table 3).

**Table 3:** Post-thaw survival and pregnancy outcomes of vitrified blastocysts derived from the anti-inhibin antibody supplementation system.

Group	No. of Vitrified Blastocysts (Replicates)	No. of Warmed Blastocysts	Recovery Rate (%)	No. of Survived Blastocysts	Survival Rate (%)	No. of Pregnancies	Pregnancy Rate (%)
Control	86 (3)	84	97.73 ± 1.14 ^a^	80	93.00 ± 0.29 ^a^	58	67.26 ± 2.33 ^b^
AIA	194 (3)	192	98.86 ± 0.57 ^a^	186	95.72 ± 0.85 ^a^	153	78.65 ± 1.57 ^a^
AIA + INHA	100 (3)	97	96.99 ± 0.13 ^a^	93	92.97 ± 1.06 ^a^	65	65.17 ± 2.06 ^b^
IgG	100 (3)	98	98.04 ± 0.98 ^a^	93	93.04 ± 0.81 ^a^	68	67.97 ± 1.21 ^b^

Note: Data are presented as mean ± SEM. Different superscripts (a, b) within the same column indicate significant differences. Survival rate refers to blastocyst re-expansion post-warming. Pregnancy rate was confirmed 60 days post-transfer. To control for recipient-related variables, all transfers were balanced across treatments using synchronized, nulliparous heifers at a single farm.

## 4. Discussion

In this study, we explored the effects of targeting inhibin on Wagyu oocytes through two independent proof-of-concept models. Using the in vivo active immunization model, we demonstrated that neutralizing inhibin effectively ameliorates oxidative stress and improves the initial metabolic status of retrieved oocytes. Oxidative stress, arising from an imbalance between ROS production and antioxidant defense, is a primary driver of developmental arrest in IVP embryos [7]. While previous studies have focused on supplementing exogenous antioxidants (e.g., melatonin, resveratrol, or glutathione) to scavenge ROS [11,12], our approach in vivo targets an upstream physiological regulator. We observed that inhibin neutralization significantly elevated the intracellular GSH/GSSG ratio and NADPH levels in OPU-derived oocytes. This finding is consistent with the role of the Activin-Nrf2 signaling axis, which has been reported to regulate antioxidant gene expression [22]. Unlike exogenous additives that may exhibit half-life limitations or concentration-dependent toxicity [23,24,25], the immunomodulation of inhibin likely creates a sustained “pro-antioxidant” follicular microenvironment. This suggests that the high levels of inhibin typically found in subordinate follicles may act as a paracrine repressor of the oocyte’s intrinsic antioxidant defense, and its removal restores the oocyte’s capacity to combat oxidative injury induced by in vitro manipulation.

Mitochondria are the hub of oocyte competence, providing the ATP required for meiotic spindle assembly [26,27,28]. A key finding of our in vivo model is the restoration of ΔΨm and distribution patterns following inhibin neutralization. Contrasting with studies that use metabolic modulators directly [29], our data suggest a physiological “release of suppression” mechanism within the ovary. The literature establishes that inhibin competitively antagonizes Activin and TGF-β signaling, pathways that are crucial for promoting granulosa cell-oocyte metabolic coupling and glucose uptake [30,31]. The removal of this “metabolic brake” likely facilitates enhanced substrate transport and mitochondrial activity, as evidenced by the significantly elevated ATP content and restored ΔΨm in the retrieved oocytes. While the precise downstream signaling cascade (e.g., SMAD2/3 or Nrf2 activation) requires further direct validation, our phenotypic findings strongly support the notion that removing inhibin’s suppressive effect successfully restores intracellular redox homeostasis and mitochondrial plasticity in the ovarian follicle [32].

While in vivo immunization validates the physiological benefits of neutralizing inhibin at the source, we utilized a completely independent in vitro model (AIA supplementation during IVM of abattoir-derived oocytes) to assess its direct impact on downstream developmental competence. Our results show that AIA supplementation significantly increased blastocyst yield and reduced apoptosis. This is comparable to the effects observed with other growth factors like GDF9 or BMP15 [33,34]. However, a unique advantage of AIA is its specificity in targeting a negative regulator. The significant reduction in TUNEL-positive cells in the AIA group mirrors the protective effects of glutathione reported in our previous work [12]. It is plausible that AIA prevents the autocrine negative feedback of inhibin secreted by cumulus cells in the culture dish, thereby maintaining the gap junctional communication required for the transfer of antioxidant precursors from cumulus cells to the oocyte.

The ultimate benchmark for the translational utility of any IVP platform relies on its capacity to generate viable pregnancies. In this regard, the 78.65% pregnancy rate obtained following the transfer of in vitro AIA-derived blastocysts indicates remarkable developmental competence. Mechanistically, this high viability can be attributed to the optimized lineage allocation achieved in vitro, characterized by a significantly higher ICM/TE ratio. Because adequate ICM cellularity is indispensable for post-implantation development and long-term fetal survival [35,36,37], we hypothesize that the inhibin-neutralized culture conditions confer a distinct “metabolic memory”. This early developmental programming likely provides the blastocyst with a superior adaptive capacity to meet the physiological and metabolic demands of the recipient uterus. Future studies should further investigate the epigenetic stability of these embryos, as oxidative stress is a known disruptor of DNA methylation patterns [38].

Finally, it is important to acknowledge certain limitations of the present study. Regarding the in vivo model, we relied on OPU oocyte yield and morphological grading as proxies for ovarian response; we did not directly measure circulating endocrine mediators (e.g., FSH) or track follicular dynamics, meaning that the exact physiological mechanisms of recruitment require further endocrine validation. Furthermore, oocytes from immunized donors were dedicated to physiological assays and not subjected to IVP; therefore, there is no direct evidence in this study that donor immunization improves blastocyst or pregnancy rates. Lastly, while the pregnancy outcomes from the in vitro AIA model are promising, pregnancy remains a multifactorial endpoint strongly influenced by recipient variables. As such, these transfer outcomes serve as descriptive indicators of embryo cryotolerance and viability, rather than absolute causal proof. Despite these limitations, the convergent evidence from both independent models robustly demonstrates that alleviating inhibin-mediated suppression is a highly effective physiological target for mitigating oxidative stress and enhancing Wagyu oocyte competence.

## 5. Conclusions

In conclusion, this study evaluated the effects of targeting inhibin on Wagyu oocytes through two independent proof-of-concept models. The in vivo active immunization model effectively increased OPU recovery efficiency and improved the initial mitochondrial bioenergetics and redox status of retrieved oocytes. Concurrently, the independent in vitro antibody supplementation model significantly enhanced blastocyst development, reduced apoptosis, and yielded blastocysts with robust post-transfer viability. Both distinct approaches highlight the importance of mitigating oxidative stress by relieving inhibin-mediated suppression. While this study focused on Wagyu cattle, the highly conserved physiological role of the inhibin-ROS axis suggests that these findings can be expanded to other large ruminant breeds. Ultimately, these distinct physiology-based strategies offer practical and effective avenues to overcome efficiency bottlenecks in the cattle breeding industry, maximizing the reproductive potential of high-value genetic resources.

## Figures and Tables

**Table 1:** Effect of active immunization against inhibin on follicular recruitment and morphological classification of collected COCs in Wagyu donors.

Group	No. of Donors (Replicates)	Total COCs Recovered	Grade A COCs	Grade A (%)	Grade B COCs	Grade B (%)	Grade C COCs	Grade C (%)	Grade D COCs	Grade D (%)
Control	30 (3)	279	98	34.99 ± 1.25 ^b^	72	25.71 ± 1.62 ^b^	56	20.26 ± 2.42 ^a^	53	29.04 ± 0.48 ^a^
INHA	30 (3)	461	211	45.66 ± 1.15 ^a^	156	33.89 ± 1.17 ^a^	55	11.98 ± 1.23 ^b^	39	8.47 ± 0.29 ^b^

Note: Data are presented as mean ± SEM. Values with different superscripts (a, b) within the same column indicate significant differences (exact *p*-values are detailed in the text). The statistical unit is the independent donor cow (n = 30 per group). COCs: Cumulus–oocyte complexes. INHA: Inhibin-immunized group.

**Table 2:** Effects of anti-inhibin antibody supplementation during IVM on oocyte nuclear maturation and in vitro embryo production parameters.

Group	No. of COCs (Replicates)	No. of MII Oocytes	Maturation Rate (%)	No. of Cleaved	Cleavage Rate (%)	No. of Blastocysts	Blastocyst Rate (%)	No. of Transferable Blastocysts	Transferable Blastocyst Rate (%)
Control	278 (3)	233	83.72 ± 1.60 ^b^	209	75.16 ± 0.29 ^b^	92	44.07 ± 0.85 ^b^	86	30.92 ± 0.19 ^b^
AIA	443 (3)	412	92.96 ± 1.04 ^a^	383	86.32 ± 2.11 ^a^	218	56.63 ± 2.36 ^a^	194	43.60 ± 2.04 ^a^
AIA + INHA	321 (3)	265	82.49 ± 1.61 ^b^	239	74.43 ± 0.66 ^b^	107	44.73 ± 0.81 ^b^	100	31.13 ± 0.64 ^b^
IgG	325 (3)	269	82.76 ± 0.88 ^b^	248	76.32 ± 0.22 ^b^	108	43.55 ± 0.51 ^b^	100	30.76 ± 0.31 ^b^

Note: Data are presented as mean ± SEM based on replicate-level means from three independent IVP sessions (n = 3). Different superscripts (a, b) within the same column indicate significant differences (exact *p*-values are detailed in the text). A higher initial number of COCs was allocated to the AIA group to accommodate subsequent destructive cellular assays. AIA: Anti-inhibin antibody group.

## Data Availability

The original contributions presented in this study are included in the article. Further inquiries can be directed to the corresponding author.
